# Insulin is necessary but not sufficient: changing the therapeutic paradigm in type 1 diabetes

**DOI:** 10.12688/f1000research.21801.1

**Published:** 2020-07-30

**Authors:** Sandra Lord, Carla J. Greenbaum

**Affiliations:** 1 Benaroya Research Institute at Virginia Mason, Seattle, WA, 98101, USA

**Keywords:** Type 1 diabetes, immunotherapy, islet autoimmunity, prevention, teplizumab, TrialNet

## Abstract

Despite the clear evidence that type 1 diabetes (T1D) begins well before hyperglycemia is evident, there are no clinically available disease-modifying therapies for early-stage disease. However, following the exciting results of the Teplizumab Prevention Study, the first study to demonstrate that overt T1D can be delayed with immunotherapy, there is renewed optimism that in the future, T1D will be treated before hyperglycemia develops. A different treatment paradigm is needed, as a majority of people with T1D do not meet the glycemic targets that are associated with a lower risk of T1D complications and therefore remain vulnerable to complications and shortened life expectancy. The following review will outline the history and current status of immunotherapy for T1D and highlight some challenges and ideas for the future. Although such efforts have been worldwide, we will focus particularly on the activities of Diabetes TrialNet, a National Institutes of Health consortium launched in 2004.

## Introduction

Nearly 100 years after its first clinical use, insulin remains the primary treatment of type 1 diabetes (T1D). Additionally, although T1D begins with asymptomatic but detectable islet autoimmunity, it is not treated until hyperglycemia begins. Certainly, there have been therapeutic improvements in T1D management, namely increasingly physiologic insulins and insulin delivery methods, continuous glucose monitoring, and closed-loop “artificial pancreas” systems. With these improvements, there have been corresponding reductions in short- and long-term complication rates. But only a minority of people living with T1D meet the hemoglobin A1c (a blood test that estimates the previous two- to three-month average glycemic control) targets that are associated with a lower risk of complications. This was suggested by 2014 data from the T1D Exchange ( https://t1dexchange.org/) in which only 17 to 23% of patients under 18 years old, 14% of those from 18 to 25 years old, and 30% of those over 25 years old had an A1c recommended by American Diabetes Association guidelines ^1^. Furthermore, T1D Exchange data from 2016 to 2018 indicate that these statistics have not improved despite the increasing use of devices ^2^. Even with adequate glycemic control, T1D management presents a financial, cognitive, and emotional strain for individuals and families. Clearly, there is an unmet need to treat T1D in earlier stages, when islet autoimmunity is apparent but before hyperglycemia begins, and it seems clear that immunotherapy will play a role. There are now five immunotherapies with demonstrated efficacy in preserving insulin secretion shortly after a T1D diagnosis. Additionally, with the results of the TrialNet Teplizumab Prevention Study ^3^, there is renewed optimism that clinical T1D can be delayed or prevented altogether.

## Type 1 diabetes begins with islet autoimmunity

**Figure 1.**
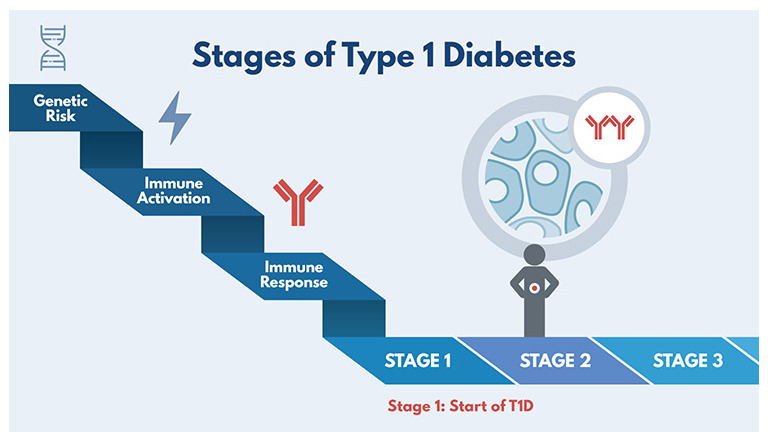
The stages of type 1 diabetes (T1D). Diabetes TrialNet was created in 2004 by the National Institutes of Health with the objective of conducting research studies to prevent T1D. It is an international network of T1D researchers who are exploring ways to prevent, delay, and slow the progression of the disease. Reprinted with permission from Diabetes TrialNet.

T1D was first categorized as an autoimmune disease over 40 years ago with the identification of islet-specific antibodies in pancreatic islets and blood ^4, 5^. Since then, both longitudinal and cross-sectional studies have described the natural history of the disease, which requires both a genetic predisposition and environmental trigger(s) and then progresses along a predictable path toward islet autoimmunity. The highest genetic risk in people of European background is conferred by HLA class II DR3/4 genes, suggesting an important role of CD4 ^+ ^T cells, although an increasing number of non-HLA risk genes have been identified ^6^. There are five well-validated autoantibodies associated with T1D: antibodies to glutamic acid decarboxylase, antibodies to insulin (insulin autoantibodies), antibodies to insulinoma-associated protein (IA-2), islet cell antibodies, and antibodies to a zinc transporter (ZnT8). Clinical T1D becomes inevitable with the development of two or more autoantibodies. This disease model described in a consensus conference ^7^ is illustrated by Diabetes TrialNet ( https://www.trialnet.org/) in Figure 1, where stage 1 T1D corresponds to two or more antibodies with normal glucose tolerance, stage 2 corresponds to two or more antibodies with abnormal glucose tolerance but still no persistent hyperglycemia, and stage 3 corresponds to a clinical diagnosis of T1D.

With the understanding that islet autoimmunity is early-stage T1D, there is greater urgency for identification of at-risk individuals. One approach is to screen for autoantibodies in those with increased genetic risk, as defined by a family history of T1D or by high-risk HLA testing at birth. But screening based on family history or genetic risk only is insufficient to identify all of those who will develop disease, as about 85% of people with T1D have no first-degree relative with T1D and only about 50% of Caucasians with diagnosed T1D have the highest-risk HLA class II DR3/4 haplotype. Another approach is to offer periodic autoantibody testing to everyone, perhaps in the pre-school years. This approach would identify most people destined to develop T1D by puberty: of this group, 64% will already have autoantibodies by age two and 95% will have autoantibodies by age five ^8^. General population screening was piloted in the Fr1da study ^9^, in which about 90,000 Bavarian children from ages two to five were screened for islet autoantibodies. As recently reported ^9^, Fr1da suggested that the risk of progression from early-stage T1D to clinical T1D is similar in the general population compared with individuals with a genetic risk of T1D. Like other studies, Fr1da found that the incidence of diabetic ketoacidosis (DKA) (3.2%) was lower at the time of clinical T1D diagnosis as compared with rates of DKA at diagnoses with usual care, which are reported to be 40 to 59% ^10, 11^. Overall, the Fr1da results support the feasibility and utility of population-wide autoantibody screening.

## Immunotherapy works and is safe

### New-onset studies

Multiple immunotherapies have been tested in new-onset T1D (within 100 days of diagnosis) with a goal of preserving remaining endogenous insulin secretion, as measured by C-peptide. C-peptide is secreted with insulin in equimolar amounts and can be used as a marker of endogenous insulin secretion after insulin therapy is started. Five immunotherapies with an acceptable side effect/safety profile have been shown to preserve insulin secretion in newly diagnosed T1D: teplizumab and otelixizumab ^12^, rituximab, abatacept, low-dose anti-thymocyte globulin (ATG), and alefacept. These agents and studies are described in Table 1. Notably, the beneficial effect of therapy on C-peptide is most apparent soon after randomization. The decline in C-peptide eventually parallels the control groups, indicating that therapy has not halted the disease. However, for several therapies, there remain significant differences between treatment arms in C-peptide level even years later. These differences may be clinically important, as multiple studies have shown the benefits of C-peptide preservation, including a lower risk of chronic complications and severe hypoglycemia even in subjects with a barely detectable C-peptide level ^13– 15^.

**Table 1.** Five selected immunotherapies with proven efficacy to preserve C-peptide at 1 and/or 2 years post randomization in phase 2 studies in new-onset type 1 diabetes (within 100 days of diagnosis).

Agent tested	Mechanism of action	Drug administration	Reference(s)
Abatacept	CTLA4 immunoglobulin (Ig), co- stimulatory blockade: disrupts antigen presentation	Monthly intravenous (iv) infusion for 2 years	25, 26
Alefacept	LFA-3 Ig: inhibition of activated T cells (primarily memory T cells)	Two courses of 12 weekly intramuscular (im) injections, separated by a 12-week pause	27, 28
Low-dose (2.5 mg/kg) anti- thymocyte globulin (ATG)	Lymphocyte depletion	One course of two iv infusions over 2 to 3 days	29, 30
Rituximab	Anti-CD20, anti-B cell: disrupts antigen presentation	One course of four weekly iv infusions	31, 32
Teplizumab (multiple studies)	Anti-CD3, inhibition of activated T cells	One course of 14 daily iv infusions. Immune Tolerance Network new-onset teplizumab study (AbATE) gave a second 14-day infusion 1 year later.	33, 34, 35

### Prevention of clinical disease

**Figure 2.**
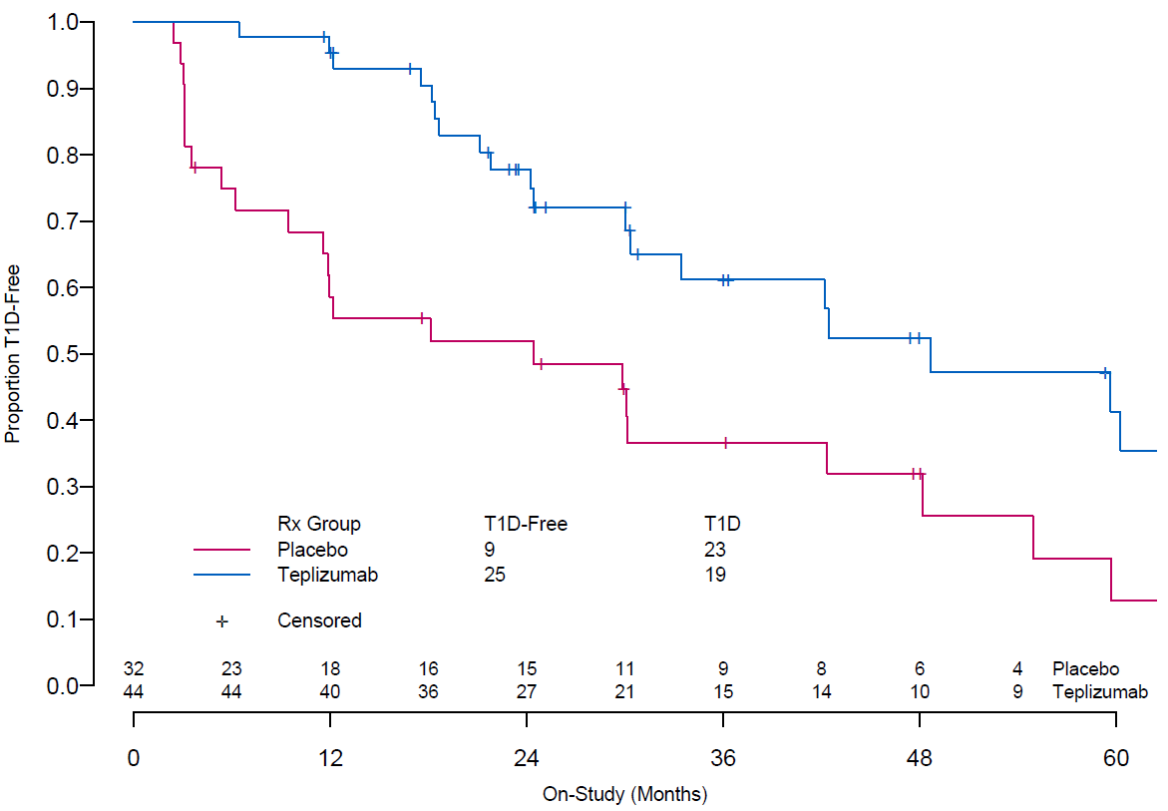
The median times to diagnosis to clinical type 1 diabetes (T1D) were 48.4 months in the teplizumab group and 24.4 months in the placebo group. This figure was reproduced from Herold *et al*. ^3^. Copyright © (2019) Massachusetts Medical Society. Reprinted with permission from Massachusetts Medical Society.

Although multiple agents have proven efficacious in new-onset T1D, disease prevention has been more elusive. In 2011, TrialNet launched the Teplizumab Prevention Study in a cohort of high-risk individuals with multiple autoantibodies and impaired glucose tolerance but without clinical T1D. Teplizumab is an anti-CD3 monoclonal antibody that does not bind the Fc receptor but does disrupt autoreactive T-cell function and may enhance regulatory T-cell function. The results of the TrialNet study, published online in the *New England Journal of Medicine* in June 2019 ^3^, provide the first evidence that clinical T1D can be delayed with immunotherapy. Seventy-six participants received 14 daily infusions of placebo or teplizumab, followed by regular monitoring with oral glucose tolerance testing. At study end, participants who received teplizumab had a median 24-month delay in clinical T1D diagnosis as compared with participants who received placebo ( Figure 2). Importantly, there was no difference in new infections between placebo- and teplizumab-treated cohorts, confirming safety outcomes from the new-onset teplizumab studies. Ongoing monitoring of participants who had not yet developed T1D at study end will provide additional information about safety, potential duration of benefit, and characteristics of long-term responders. ****

The Teplizumab Prevention Study provides a framework for testing additional immunotherapies with proven efficacy in new-onset T1D prior to clinical disease. TrialNet is testing the CTLA 4-Fc fusion protein abatacept in individuals with multiple autoantibodies to see whether it can delay clinical T1D ^16^ and will soon launch a study of the anti–B cell agent rituximab followed by abatacept, also in an at-risk population ^17^. This combination study is described below (in the ‘Combination or sequential use of therapies may provide therapeutic synergy’ section). Another TrialNet study under consideration will investigate whether clinical T1D can be delayed in a high-risk population with multiple autoantibodies using low-dose ATG.

Whereas teplizumab, abatacept, rituximab, and ATG have been or are being considered for T1D prevention following their positive outcomes in recently diagnosed individuals, other therapies can be tested for T1D prevention or progression without being tested first in new-onset T1D. For example, previous trials have tested parenteral, nasal, and oral insulin ^18– 21^; nicotinamide ^22^; and hydrolysed casein cow’s milk formula ^23, 24^. Although the primary outcomes of these agents were negative, these therapies are known to be safe and have scientific rationale for use in early-stage T1D. The Global Platform for the Prevention of Autoimmune Diabetes is a multicenter European initiative to provide a framework for T1D primary prevention trials such as POInT (Primary Oral Insulin Trial) ^22, 36^. POInT will test whether oral insulin can delay or prevent the development of autoantibodies and T1D in genetically high-risk infants. Another ongoing prevention study is the TrialNet Hydroxychloroquine Prevention study, which is investigating whether hydroxychloroquine can delay or prevent progression to impaired glucose tolerance or clinical T1D (or both) in subjects with multiple autoantibodies and normal glucose tolerance. Hydroxychloroquine is a well-tolerated and inexpensive agent used historically for the treatment of malaria and currently for the treatment of rheumatoid arthritis and lupus. Although it has not been used in T1D, it has several immune properties anticipated to have beneficial effects in pre-clinical T1D, namely decreased cell activation, antigen presentation, and autoantibody production ^37^. An additional TrialNet prevention trial under development will test methyldopa, which is an alpha-adrenergic blocker used clinically for pregnancy-induced hypertension. Interestingly, it has a crystal structure that fits in the cleft of the major histocompatibility complex (MHC) class II molecule HLA-DQ8 that binds insulin peptides. Insulin is an early immune target in T1D; moreover, insulin autoantibody positivity has been associated with HLA DQ8 positivity ^38, 39^. In a small study of HLA-DQ8 ^+^ patients with recent-onset T1D, methyldopa was shown to specifically block insulin peptide binding to HLA-DQ8 and to decrease inflammatory T-cell responses to insulin ^40^. TrialNet aims to launch a similar study testing the effects of methyldopa on antigen presentation in a DQ8 ^+^ at-risk population of children and adults with insulin antibodies ^41^.

## How to personalize immunotherapy in type 1 diabetes

As demonstrated in new-onset studies, immunotherapy can preserve insulin secretion after diagnosis in some patients, but a significant portion of drug-treated patients have a C-peptide decline similar to that of placebo-treated patients. This was seen in the Immune Tolerance Network new-onset teplizumab study (AbATE), in which 45% of teplizumab-treated subjects maintained their C-peptide level at two years but 55% of teplizumab-treated individuals appeared indistinguishable from placebo-treated controls ^42^. Further work is needed to predict and monitor response to therapy and to decide when and whom to treat. The following section describes some early observations.

### Children respond well to therapy

Although we no longer call T1D “juvenile” diabetes, many people with T1D are diagnosed as children and will live with T1D for decades. Several studies in new-onset T1D suggest that it may be easier to demonstrate the benefits of immunotherapy in children as compared with adults. For example, the positive result seen in both the rituximab and abatacept new-onset studies primarily reflected the response seen in children ^31, 32, 43^. Since these studies were not designed to compare effects between adults and children, this does not imply that therapy does not work in adults. Indeed, age was not identified as a factor of response in the Teplizumab Prevention Study. However, it is known that children lose insulin more rapidly and completely after diagnosis as compared with adults ^44^, suggesting more aggressive immune activity in children. In fact, 35% of individuals with T1D diagnosed after age 18 have detectable C-peptide 10 to 19 years after diagnosis as compared with only 9% of people whose T1D is diagnosed before age 18 ^45^. Further evidence comes from insulitic profiles of pathology specimens ^46^ and from RNA sequence data ^47^ which support the concept that age affects immune phenotype in T1D. Taken together, these observations suggest that although both children and adults benefit from early intervention, the benefits to children may be more apparent. Moreover, in children, several years’ delay in clinical diagnosis is clinically important as adults tend to manage the demands of T1D better than teens and children do. Furthermore, because the incidence of T1D may decrease with age, a “pause” in immune activity might allow tolerance to develop naturally.

### Genetic characteristics

HLA genes are the largest contributors to T1D risk; however, other genes encode proteins with direct or indirect immune effects on T1D pathophysiology. Interestingly, HLA genes appear to contribute primarily to the risk of autoantibody development with little contribution to progression once multiple antibodies are present ^48– 50^. Although the data evaluating the role of non-HLA genes are much less robust, some evidence suggests that non-HLA genes may contribute to progression from antibody positivity to overt T1D ^51^. There are also hints that HLA genes may affect response to therapy. For example, in the TrialNet Teplizumab Prevention Study, teplizumab-treated participants who were HLA-DR3 ^−^/DR4 ^+^ had a delay in T1D diagnosis whereas DR3 ^+^/DR4 ^−^ individuals progressed similarly to individuals who received placebo.

### Immune characteristics

Whereas baseline immune characteristics have not yet been associated with response to therapy, post-treatment immune phenotypes are more commonly identified in responders as compared with non-responders. This was seen in both AbATE and the TrialNet Teplizumab Prevention Study, where individuals with markers of T-cell exhaustion (associated with prolonged antigen stimulation and loss of CD8 effector function) were shown to have responded better to teplizumab treatment ^3, 33^. Additionally, there are clues that immunotherapy may be most effective during periods of robust immune activity, as suggested by the Teplizumab Prevention Study, in which participants who began the study with a below-median C-peptide level (perhaps a marker of more active disease) responded better to teplizumab treatment as compared with those who started treatment with a C-peptide level above the median ^3^. Such markers might be used to identify therapeutic windows for treatment both initially and during a relapse.

## Looking to the future

Despite significant improvements over the last 100 years, most individuals with T1D do not meet therapeutic targets and remain vulnerable to complications, suggesting that current insulin delivery technologies are not enough. The T1D disease model has changed: islet autoimmunity, as measured by two or more T1D-specific autoantibodies, is now considered the first stage of T1D. Early treatment with immunotherapy to prevent tissue destruction and loss of function is already the standard of care in other autoimmune conditions, such as rheumatoid arthritis or multiple sclerosis. It is likely that immune therapy prior to and/or shortly after clinical diagnosis will be part of routine care in T1D. Moreover, though not addressed in this review, approaches to directly target beta cells may also become part of T1D therapy.

### Consider alternative study designs to speed study enrolment and completion while decreasing cost

Analysis of surrogate endpoints earlier in the development of T1D might decrease the time needed to evaluate treatment efficacy. Given the inevitability of clinical T1D once there are two or more autoantibodies, the development of islet autoimmunity might be used as an intermediate endpoint for primary prevention studies. Similarly, because islet autoimmunity with normal glucose tolerance progresses to islet autoimmunity with abnormal glucose tolerance and then to clinical T1D, the development of abnormal glucose tolerance is being used as a study endpoint in studies testing therapy to delay progression from islet autoimmunity to T1D. This approach is being tested in the TrialNet Abatacept in Prevention and Hydroxychloroquine in Prevention studies, described earlier ^16, 37^. Adaptive trial designs ^52^, studies with mechanistic or composite endpoints ^53^, or single-arm studies might be conducted as alternatives to the “gold standard” placebo-controlled trial, both to facilitate participant recruitment and to reduce cost. With considerable amounts of T1D natural history data available, it is feasible that existing data sets may be used in a single-arm study to obviate the need for a placebo cohort.

### General population screening will be needed to identify most people with islet autoimmunity

As outlined earlier, this might be accomplished with genetic testing at birth followed by periodic autoantibody testing in those who have high-risk genes or a family history of T1D or with periodic autoantibody testing for everyone. However it is accomplished, general population screening is costly and will require considerable effort in education and counselling for those who are identified as being at risk and have no experience with T1D. Screening family members for T1D risk can be anxiety-provoking, as learning that oneself or one’s child is at high risk creates stress and uncertainty and may create discrimination or alter life plans. Despite this, studies suggest that the initial anxiety associated with screening dissipates with repeated testing ^54^. Further work found that when T1D is diagnosed through screening efforts, there is less stress than in families diagnosed without risk screening ^55^. The Fr1da study suggests a similar pattern in those without T1D in their families: parental stress was higher after receiving a diagnosis of early-stage T1D as compared with those who received negative test results but this declined in the year after testing ^9^. Moreover, as reported in Fr1da, parental stress was lower in the cohort whose children were diagnosed with early-stage asymptomatic T1D compared with a cohort from the DiMelli study ^56^ whose children were diagnosed with clinical T1D without prior staging.

### Chronic immunotherapy may be necessary

Despite early optimism that a short course of immunotherapy would produce a durable or permanent remission through restoration of self-tolerance, this has not been the case, nor has it been true in any other autoimmune disease. It is likely that chronic or intermittent treatment will be needed to preserve beta-cell function and prevent progression of disease ^57^. This strategy has not been tested in T1D but becomes increasingly feasible as new and safer immunotherapies become available for the long-term therapy of other autoimmune diseases.

### Combination or sequential use of therapies may provide therapeutic synergy

There is a precedent for combination immunotherapies in other immune-mediated diseases, such as the combination of methotrexate and etanercept in rheumatoid arthritis ^58^ or azathioprine plus infliximab in Crohn’s disease ^59^. TrialNet aims to launch a study in a high-risk population with multiple autoantibodies and impaired glucose tolerance that will evaluate the effect on disease progression with rituximab followed by abatacept. The combination treatment was suggested by findings from each of the abatacept and rituximab monotherapy new-onset trials. In the abatacept study, participants with a high B-cell signature six months after the start of therapy were less likely to have responded to abatacept treatment ^60^. Similarly, in the rituximab study, participants with a high activated T-cell signature six months after rituximab treatment were less likely to have responded to rituximab therapy ^61^. In the planned TrialNet combination study, four weekly treatments with rituximab will deplete CD20 ^+^ B cells. Then four months later, prior to B-cell recovery, abatacept treatment will start and continue for two years. Abatacept is expected to interfere with T-cell help necessary to B-cell recovery, thus precluding recovery of autoreactive B cells.

### Lower cost and greater convenience of immunotherapy are anticipated

Although immunotherapy is expensive and inconvenient, so is a lifetime of T1D. A 2019 report by the Health Care Cost Institute ( https://www.healthcostinstitute.org/research/publications/entry/spending-on-individuals-with-type-1-diabetes-and-the-role-of-rapidly-increasing-insulin-prices) estimates that the 2016 average annual health-care spending for a person with T1D was $18,494 and this does not include indirect costs. Although the specific costs of each approach are currently unknown, it is expected that oral and subcutaneous versions of immunotherapies that can be administered at home will increase the feasibility of immunotherapy for T1D and that the increasing availability of “bioequivalent” or generic versions will decrease cost. Thus, we are reaching a point where even long-term preventative immunotherapy may be more cost-effective than lifelong T1D treatment.

### Greater advocacy is needed

To shift the T1D treatment paradigm, a multifaceted approach will be needed: to understand the ongoing burden of T1D despite therapeutic successes; to normalize the new T1D disease model, that is, that T1D begins long before hyperglycemia; and to recognize the potential for immunotherapy to modify the underlying disease. People not affected by T1D (including clinicians) may be under the impression that T1D can be managed with minimal effort and minimal risk. This impression ignores the evidence that a majority of people living with T1D do not meet recommended therapeutic targets. As a result, people with T1D (and type 2 diabetes) may apologize for their disease management rather sharing their daily struggles with the disease, including the cost and mental/emotional burdens. In addition to the unmet need for relief from hyperglycemia, greater awareness that islet autoimmunity is a precursor to clinical T1D is needed. With this understanding, it seems natural to shift the therapeutic paradigm from treating hyperglycemia to preventing hyperglycemia. Although immunotherapy is not yet ready for clinical use in T1D, with the results of the Teplizumab Prevention Study—the first study to show that clinical T1D diagnosis can be delayed with immunotherapy—the possibility seems closer than ever.
